# A Low-Profile Dual-Polarized High-Gain Low Cross-Polarization Phased Array for Ku-Band Satellite Communications

**DOI:** 10.3390/s25133986

**Published:** 2025-06-26

**Authors:** Yuhan Huang, Jie Zhang, Xiuping Li, Zihang Qi, Fan Lu, Hua Jiang, Xin Xue, Hua Zhu, Xiaobin Guo

**Affiliations:** 1 Beijing Institute of Spacecraft System Engineering, Beijing Engineering Research Center of EMC and Antenna Test Technology, Beijing 100094, China; h18811409295@163.com (Y.H.); fan8736@pku.edu.cn (F.L.); jianghualtt@163.com (H.J.); xuxwindy@163.com (X.X.); 2 School of Electronic Engineering, Beijing University of Posts and Telecommunications, Beijing 100876, China; qizihang@bupt.edu.cn (Z.Q.); guo_xb@163.com (X.G.); 3 State Key Laboratory of Information Photonics and Optical Communications, Beijing 100876, China; 4 Key Laboratory of Universal Wireless Communications of Ministry of Education, Beijing 100876, China; 5 Beijing Key Laboratory of Work Safety Intelligent Monitoring, Beijing 100876, China; 6 Key Laboratory of Optoelectronic Devices and Systems of Ministry of Education and Guangdong Province, College of Physics and Optoelectronic Engineering, Shenzhen University, Shenzhen 518060, China; judy-cool@163.com

**Keywords:** dual-polarized, phased array, Ku-Band satellite communications, shared-aperture, nonuniform subarray, differential-fed, substrate integrated coaxial line

## Abstract

A low-profile dual-polarized shared-aperture phased array antenna is proposed for Ku-band satellite communications in this paper. The stacked octagonal patches loaded with Via-rings are proposed as dual-polarized shared-aperture radiation elements, with the characteristics of wide impedance bandwidth, high gain, and weak coupling. Furthermore, innovative minimized three-port ring couplers are utilized for the differential-fed antenna array, further suppressing the cross-polarization component. Substrate integrated coaxial line (SICL) and microstrip line (MS) feed networks are employed for the excitation of transmitting band (Tx) horizontal polarization and receiving band (Rx) vertical polarization, respectively. The non-uniform subarray architecture is optimized to minimize the sidelobe levels with the reduced number of transmitter and receiver (T/R) radio frequency phase-shifting modules. As proof-of-concept examples, 16 × 24 and 32 × 24 array antennas are demonstrated and fabricated. The measured impedance bandwidths of the proposed phased array antennas are around 21.1%, while the in-band isolations are above 36.7 dB. Gains up to 29 dBi and 32.4 dBi are performed by two prototypes separately. In addition, the T/R phase-shifting modules are utilized to validate the beam-scanning characteristic, which is of value for dynamic satellite communications.

## 1. Introduction

Ku-band satellite communication (SATCOM), with the superiority of high throughput and comprehensive coverage, is drawing more and more attention to dynamic inter-satellite and satellite-to-earth communications [1,2]. Phased array antennas have been utilized in the SATCOM system due to their flexible beam controlling and high communication capacity. Taking into account the capability requirement and launch cost of satellite carrier platforms, a spaceborne phased array antenna should be characterized by high-gain, lightweight, and low-profile performances.

Phased array antennas have the potential to provide flexible beam reconfigurability, such as coverage area [3,4,5], sidelobe level [6,7], and beam scanning [8,9]. Research on the interconnection between antennas and phase-shifter circuits has been discussed. Multilayer package substrates made of high-temperature co-fired ceramic (HTCC), low-temperature co-fired ceramic (LTCC), printed circuit board (PCB), thin film, or organic high-density interconnect (HDI) have been demonstrated for phased array antennas. In [8], a phased array antenna embedded in a flip-chip ball grid array (BGA) package with 11 layers of LTCC substrates is proposed. However, poor thermal conductivity and uncontrollability during the firing process of an LTCC are inevitable. Furthermore, massive adoption of transmitter/receiver (T/R) modules is challenging for process cost and complexity. As a new generation to reduce T/R modules, the subarray scheme is proposed as an effective alternative [10,11]. In [10], subarrays are utilized in a 2D phased array scheme with up to 60% reduction in the number of phase shifts while keeping sidelobes lower than −15 dB.

In addition, various researchers have sought for high-gain antennas in recent years: their methods have included waveguide [12,13,14], metasurface [15,16], and microstrip patch [17,18,19] antennas. In [13], an air-filled waveguide-fed magneto-electric (ME) antenna array is investigated and fabricated with 3D printing technology. The measured gain results are up to 28.5 dBi, but the complicated process, heavy weight, and low integration with the back-end RF circuit are disadvantages for the SATCOM application. The quad-band reflectarray antenna (RA) in [15] is proposed for the Ku- and Ka-bands. However, the aperture efficiency at the Ku-band is decreased by the overlapping radiation elements and frequency-selective surface (FSS). In addition, the multiple complex feeding system imposes some challenges to the overall profile and fixed installation. As a proven and effective generation for antenna array design, the patch antenna is an alternative to the above two types of antennas with a low profile and ease of integration. It should be noted that the maximum gain is restricted by the feed network [20]. In terms of the typical microstrip line (MS), high loss and leakage radiation are unavoidable [21,22]. Although the insertion loss of the substrate integrated waveguide (SIW) is reduced, the bandwidth is limited by the cut-off frequency of the dominant mode [23]. A substrate integrated coaxial line (SICL) is better than the above two structures and has better transmission features. The transmitted intrinsic TEM mode of SICL has the advantages of wideband single-mode operation, low loss, and interference-free performance.

In addition, differential-fed schemes have been widely proposed in the antenna array design due to their features of low cross-polarization level, symmetric radiation patterns, and leakage wave suppression. In [24], two types of 2 × 2 antenna array are proposed. One is fed with dual out-of-phase feeding probes, and the other is constructed with differential pairs of single-end antennas. The measured results of cross-polarization are about −17 dB at the boresight for the two proposed antennas. Compared with the latter antenna, the former has worse gain performance due to massive losses caused by the bulky feed network. In addition, a 4 × 4 antenna array based on a differential SIW feeding network is designed in [25]. The H-shaped four-way power divider with equal amplitude and 180° out-of-phase output signals formed with five LTCC layers is fed by rectangular waveguide transition. However, the measured cross-polarization levels are higher than the simulated results because of the slight shift of the coupling slot.

In this paper, a dual-polarized differential-fed phased array patch antenna with high gain, low sidelobes, and light weight is proposed. The 1D phased array is grouped into non-uniform subarrays along the direction of beam scanning with the suppression of sidelobe levels. Horizontal polarization radiation at the transmitting band (Tx, 14–14.5 GHz) and vertical polarization radiation at the receiving band (Rx, 12.25–12.75 GHz) share slot-coupled octagonal patches loaded by Via-rings. In addition, minimized three-port ring couplers are designed for differential feeding and further suppress the cross-polarization level. Two prototypes based on the SICL and MS feeding structure are demonstrated and fabricated. To the best of the authors’ knowledge, this is one of the few dual-polarization phased array antenna designed with T/R RF phase-shifting modules in recent years.

The rest of this paper is structured as follows. The antenna element and 4 × 4 subarray are presented in Section 2. Section 3 studies the 16 × 24 array design and measurement. After that, the 32 × 24 phased arrays and related discussions are elaborated in Section 4. Finally, conclusions are drawn in Section 5.

## 2. Design of Antenna Element and Feed Networks

**Figure 1:**
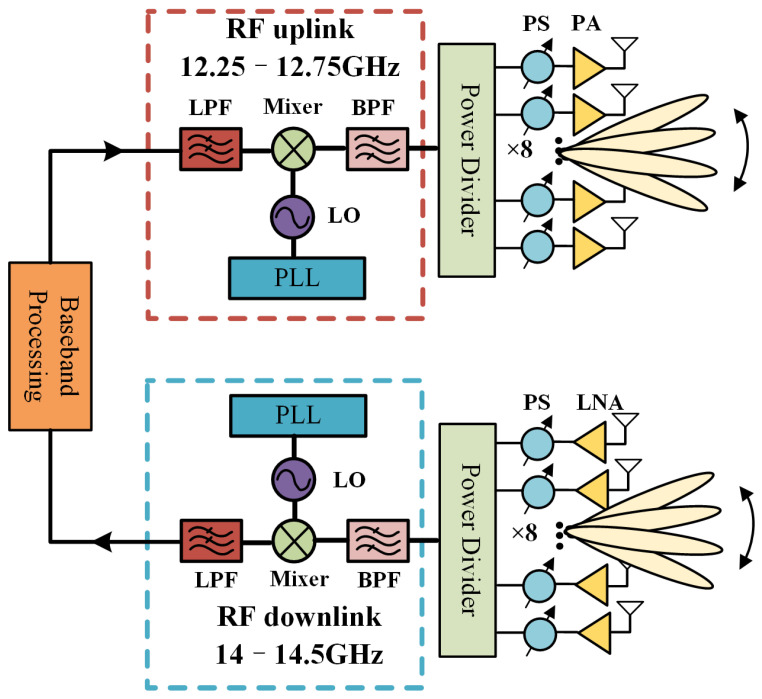
System diagram of the Ku-band satellite communication.

**Figure 2:**
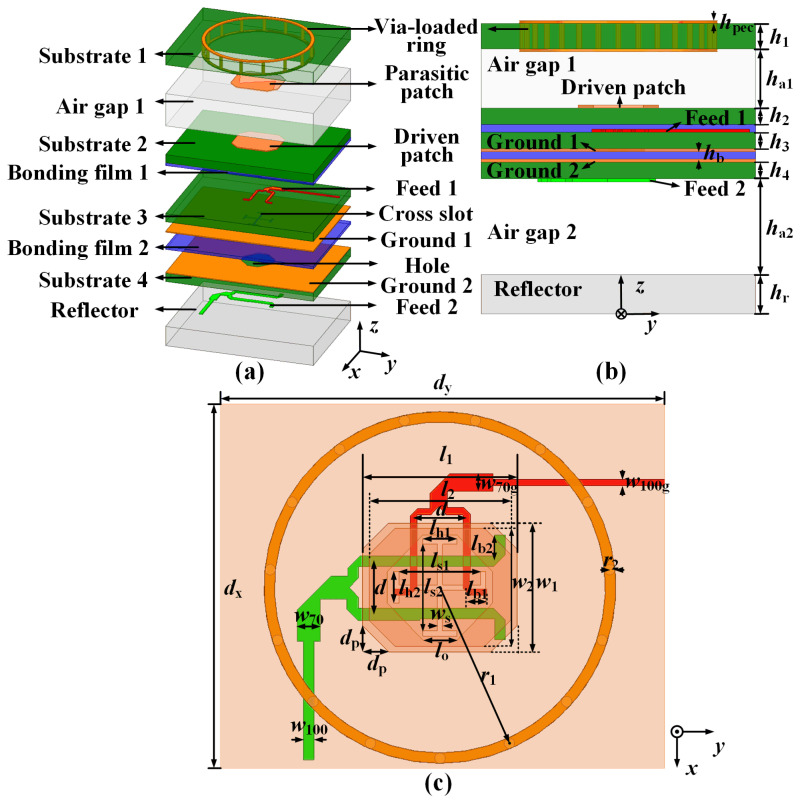
Configurations of the proposed antenna element: (**a**) Exploded view. (**b**) Side view. (**c**) Top view.

The system diagram of the Ku-band satellite communication is given in Figure 1, in which two active phased arrays are utilized for beam scanning. Figure 2 shows the configuration of the proposed antenna element, which consists of four dielectric substrates, two Bonding films, two Air gap layers, and seven metal layers. An RS300 dielectric substrate ($\varepsilon_{r1}=2.94$, $\tan\delta_{1}=0.001$) is employed as Substrates 1–4, due to its performances of low gas output, low dielectric constant thermal stability coefficient, and low thermal expansion coefficient, applying to the space SATCOM application. The dielectric material of the foam layer is ROHACELL®HF51 ($\varepsilon_{r3}=1.08$, $\tan\delta_{3}=0.0021$ at 10 GHz), serving as the Air layer. A metal Reflector is proposed to provide support and protection. In addition, a bonding pre-preg layer (PP) RLP30 ($\varepsilon_{r2}=3$, $\tan\delta_{2}=0.002$) with a thickness of 0.1 mm is adopted to bond Substrates 2–4. The thickness of the metal layers—including Ground 1, Ground 2, Feed 1, Feed 2, and Driven patch—is uniformly designed as 0.5 oz (17.5 μm). The detailed dimensions of the proposed element are listed in Table 1.

Specifically, the dual-polarized dual-band antenna element spacing is designed as $0.665\lambda_{01}\times 0.77\lambda_{02}$ ($\lambda_{01}$ is the free space wavelength at 14.25 GHz and $\lambda_{02}$ is the free space wavelength at 12.5 GHz). The seven metal layers from bottom to top are Feed 2, Ground 2, Ground 1, Feed 1, Driven patch, Parasitic patch, and Via-loaded ring. The first four layers are located on Substrates 2–4, and the last two layers are printed in Substrate 1. In detail, Feed 1 and Feed 2 are employed as the feed-line for dual-polarization operation at the Tx and Rx bands, respectively. The I-shaped slot is introduced for the slot-coupling excitation with broad bandwidth performance. Furthermore, Ground 1 and Ground 2 between the two above feed networks are introduced to reduce the coupling between two dual-polarized ports and then to enhance the port-to-port isolation. The slot-coupled octagonal Driven patch is located on the top layer of Substrate 2. The patch is symmetric along the *x*- and *y*-directions, which guarantees the shared-aperture radiation of the dual-polarized waves. An Air gap is added between Substrate 1 and Substrate 2 to enhance the broadband characteristic. In addition, an octagonal Parasitic patch on the bottom layer of Substrate 1 and the Via-loaded ring embedded in Substrate 1 is utilized to increase the gain performance and decrease the coupling between the antenna elements.

**Figure 3:**
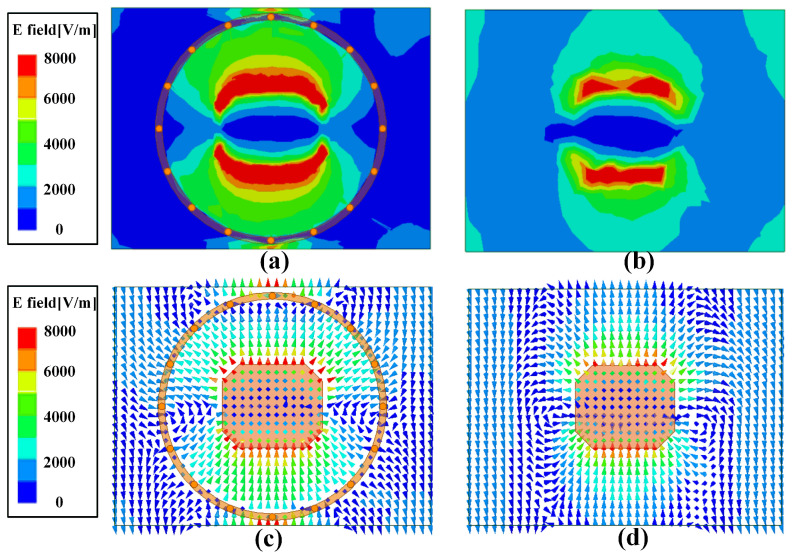
Comparison of electric field distributions with and without the Via-loaded ring in Substrate 1: (**a**,**c**) Electric field amplitude and vector distribution with the Via-loaded ring; (**b**,**d**) Electric field amplitude and vector distribution without the Via-loaded ring.

The electric field distributions in Substrate 1 of the antenna element with and without the Via-loaded ring are shown in Figure 3 for comparison. It can be seen from Figure 3a that the electromagnetic wave of the element is restrained by the Via-loaded ring as compared with that in Figure 3b, leading to weak coupling between the adjacent elements. In addition, the low cross-polarization performance is enhanced due to the octagonal patch. The electric vector of the element in Figure 3c shows that the radiations around the edges on the yoz-plane are out of phase. Therefore, the cross-polarization component is suppressed around the corners of the patch antenna.

**Figure 4:**
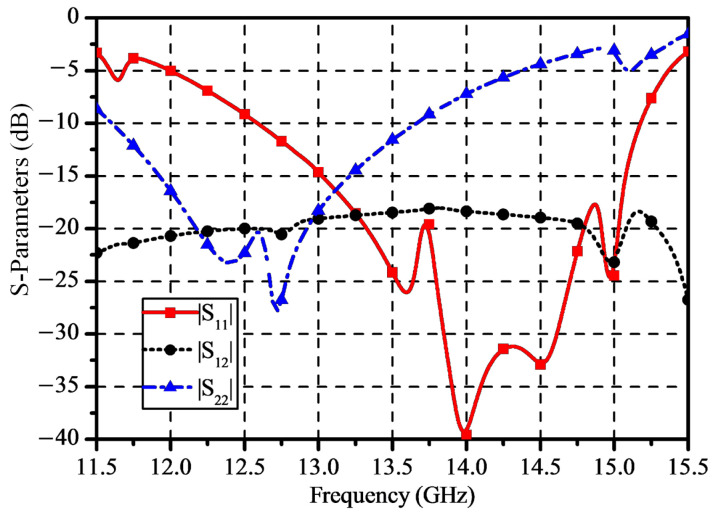
Simulated S-parameters of the proposed antenna element.

In addition, the simulated S-parameters of the element are plotted in Figure 4. The impedance bandwidth for |$S_{11}$| < −15 dB is 14.7% (from 13 GHz to 15.1 GHz) for Port 1 and 10.8% (from 11.9 GHz to 13.25 GHz) for Port 2, and the isolation results are above 17 dB in the operating bands. Therefore, the proposed dual-polarized dual-band antenna element with shared-aperture radiation has the advantages of light weight and wide band, indicating that it is valuable for the SATCOM application.

The massive adoption of T/R modules in phased arrays has an adverse impact on the cost and complexity of SATCOM applications [9]. In this paper, a non-uniform subarray architecture is adopted for beam scanning. Instead of arranging a uniform planar antenna array, a sequence of subarrays with different groupings is utilized to implement the performances of low-sidelobe-level and beam-forming.

**Figure 5:**
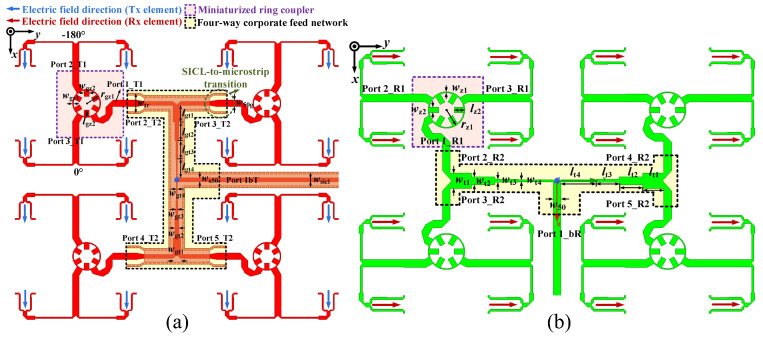
The feed networks of the 4 × 4 antenna subarray: (**a**) Tx-band feed network. (**b**) Rx-band feed network.

The Tx- and Rx-band feed networks of a 4 × 4 subarray are illustrated in Figure 5a,b, respectively. Specifically, a differential feed scheme based on a miniaturized ring coupler is adopted for the excitation of adjacent elements. The out-of-phase currents can suppress the unwanted leakage radiation and the cross-polarization component. A four-way corporate feed network is responsible for consistency in the amplitude and phase of the 4 × 4 antenna array. Furthermore, the dielectric cover effect introduced by Substrate 2 increases the dielectric loss of the Tx-band feed network (i.e., Feed 1).

**Figure 6:**
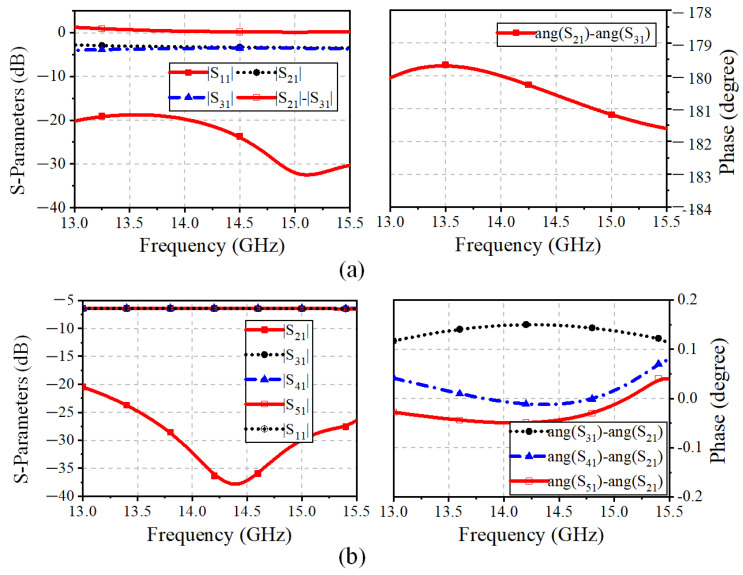
Simulated S-parameters of Tx-band feed network: (**a**) Miniaturized ring coupler. (**b**) SICL-based four-way corporate feed network.

**Figure 7:**
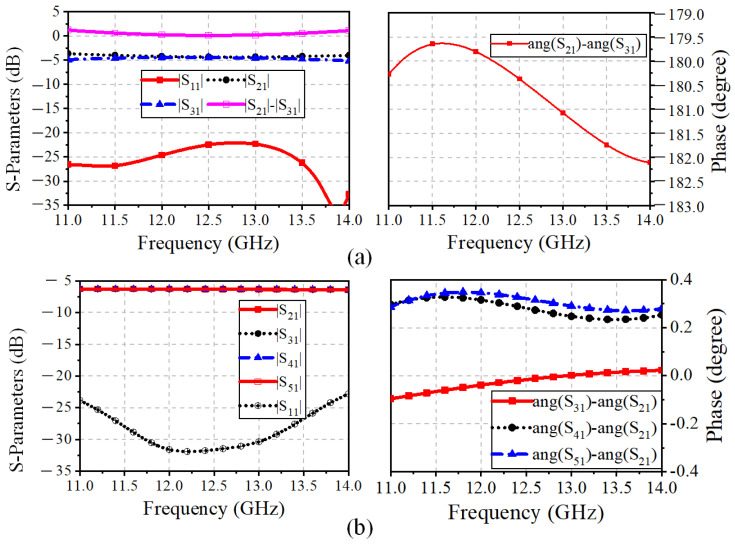
Simulated S-parameters of Rx-band feed network: (**a**) Miniaturized ring coupler. (**b**) Four-way corporate feed network.

Therefore, an SICL structure with low attenuation is adopted for the feed network design at Tx band, and the metallic Vias of the SICL are embedded in Substrate 2 and Substrate 3. The Rx-band feed network is printed on the bottom layer of Substrate 4, composed of the planar MS structure. The detailed parameters of the Tx- and Rx-band feed networks are shown in Table 2:

The configurations of the differential feed networks based on the three-port ring coupler are illustrated in the purple box in Figure 5. The three-port coupler employs a miniaturized ring MS structure. In detail, Port 1_T1 (Port1_R1 for the Rx-band feed network) is responsible for the Tx-band input signal, and Port 2_T1 and Port 3_T1 (Port 2_R1 and Port3_R1) are utilized for feeding the adjacent elements with a 180° phase difference. A branch-loaded transmission line with compact size and low complexity is proposed and implemented to replace the traditional quarter-wavelength transmission line between the differential output ports. In addition, the simulated S-parameter results of the three-port ring couplers of the Tx and Rx-bands feed networks are shown in Figure 6a and Figure 7a, respectively. The simulated |$S_{11}$| is less than −20 dB between 13.5–15 GHz and 11.5–13.0 GHz. The amplitude and phase differences between the two output ports are less than 0.7 dB and 0.25°. In this way, the proposed three-port ring couplers are responsible for achieving the differential feed with the compact structure and suppressing the cross-polarization level.

Furthermore, the four-way corporate feed networks for each polarization are aligned orthogonally, which is indicated in the yellow box in Figure 5. The Tx-band feed network is composed of T-junctions, microstrip-to-SICL transition, and an SICL-based multi-order quarter-wavelength impedance matcher. Similar to the Tx-band feed network, the Rx-band quarter-parallel feed network comprises T-junctions and a multi-order impedance matcher based on the MS structure. The simulated results of the impedance bandwidth are presented in Figure 6b and Figure 7b. It can be seen that the |$S_{11}$|of less than −20 dB are covering the Tx band and Rx band separately, while the amplitude difference and the phase difference between the output ports are less than 0.06 dB and 0.15°, respectively.

## 3. The 16 × 24 Antenna Array

**Figure 8:**
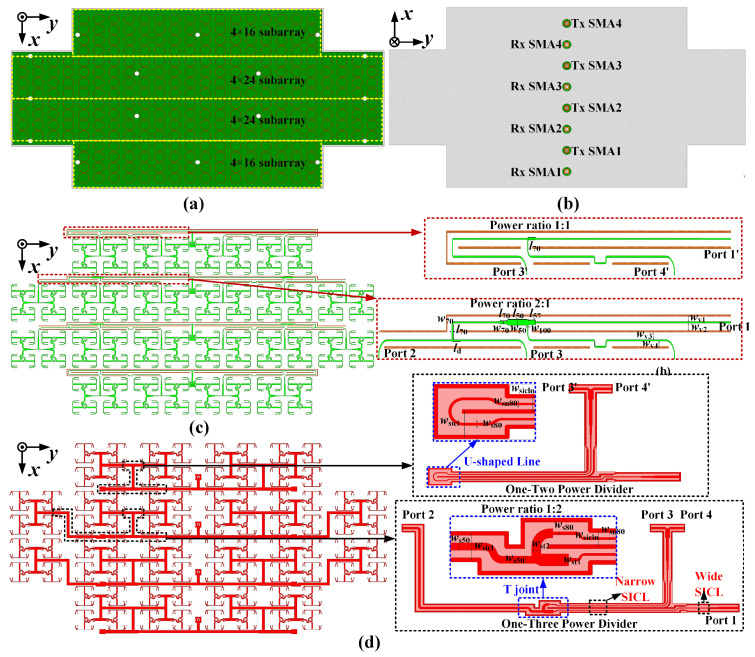
Configurations of the proposed 16 × 24 antenna array. (**a**) Top view. (**b**) Bottom view. (**c**) Rx-band feed network. (**d**) Tx-band feed network.

With the use of the 4 × 4 subarrays, a 16 × 24 antenna is designed and fabricated. The configurations of the 16 × 24 non-uniform phased array are presented in Figure 8. For the purpose of elements feeding under the equal power division radios without phase difference, the one–three powder dividers and one–two power dividers shown in Figure 8c,d are introduced.

In detail, the metal Via walls of the Rx-band one–three power divider embedded in Substrate 4 are proposed to reduce the coupling between the adjacent MS lines aligned in parallel. Furthermore, Ground 2 on the upper layer of Substrate 4 is placed between the PP layer and the Rx-band feed network, which can effectively suppress the surface wave and transmission loss. Meanwhile, the metallic Vias of the Tx-band SICL feed line are embedded in Substrates 2 and 3. The feeding structures with a wide–narrow–wide–narrow–wide SICL for the Tx-band one–three power divider contribute to achieving the wide impedance bandwidth, and the shared metallic Vias between the adjacent narrow-SICL structures are responsible for the suppression of the coupling with a miniaturized feed network design. In addition, compared with the one–three power divider, only the T-junction for feeding the additional port is replaced with a U-shaped line for the one–two powder divider, ensuring in-phase excitation.

**Figure 9:**
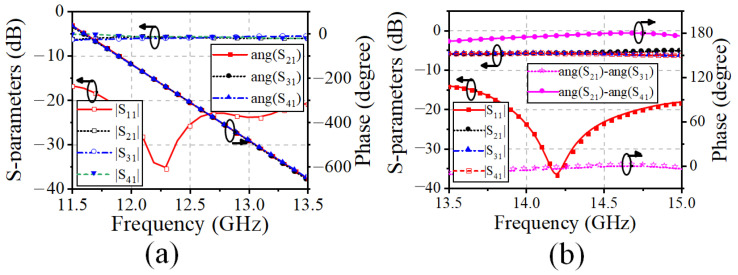
Simulated S-parameters of the one–three corporate feed network: (**a**) Rx-band feed network. (**b**) Tx-band feed network.

The simulated S-parameters of the one–three corporate feed networks are shown in Figure 9. The input port of the one–three parallel feed network with |$S_{11}$| <−20 dB is obtained at the Rx band, and the transmission coefficients of output ports 2, 3, and 4 (i.e., |$S_{12}$|, |$S_{13}$|, and |$S_{14}$|) approximately equal −5.9 dB, with an amplitude difference less than 0.5 dB. Meanwhile, the simulated magnitude and phase differences between the output port are less than 0.1 dB and 6° at the Tx band. It should be noted that the SICL-to-microstrip transition eliminates the 180° phase difference and then keeps the in-phase excitation for the proposed antenna array.

**Figure 10:**
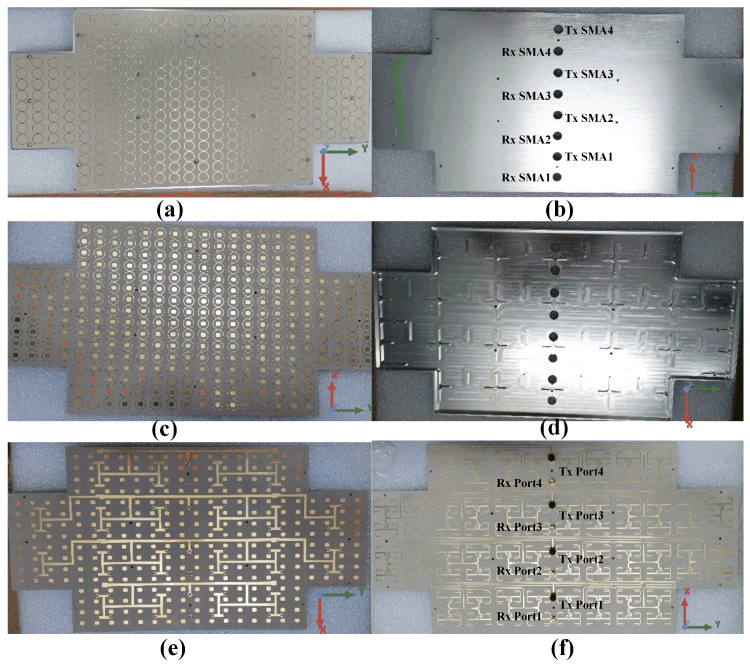
Photographs of the fabricated 16 × 24 antenna array: (**a**) Top view. (**b**) Bottom view. (**c**) Bottom view of Substrate 1. (**d**) Top view of metal reflection cavity. (**e**) Top view of Substrate 2. (**f**) Bottom view of Substrate 4.

**Figure 11:**
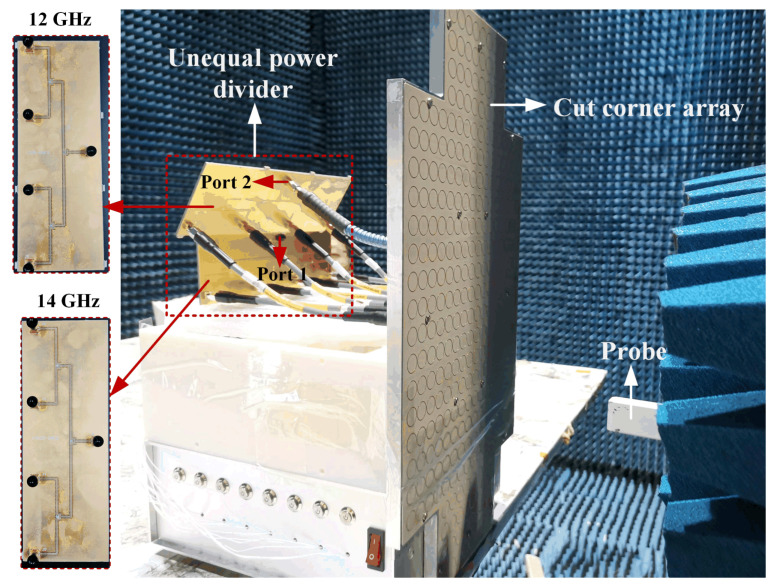
The measurement setup of the 16 × 24 antenna array in the near-field anechoic chamber.

The fabricated 16 × 24 antenna is illustrated in Figure 10. The proposed antenna arrays are fabricated by printed circuit board (PCB) and computer numerical control (CNC) machining technology. A metal Reflector cavity consisting of rectangular and cross-shaped supporting metal blocks is introduced as the container for the antenna array, machined by aluminum plate and treated by conductive oxidation. As shown in Figure 10d, these metal blocks can serve as a supporting structure between the antenna array surface and the metal Reflector. Four Tx SMA ports and four Rx SMA ports on the metal Reflector are used to connect with the T/R RF modules. Furthermore, the S-parameters are obtained by Keysight N5247B. The radiation patterns and gains are measured by the planar near-field anechoic chamber technology, and the photograph of the measurement setup is shown in Figure 11.

**Figure 12:**
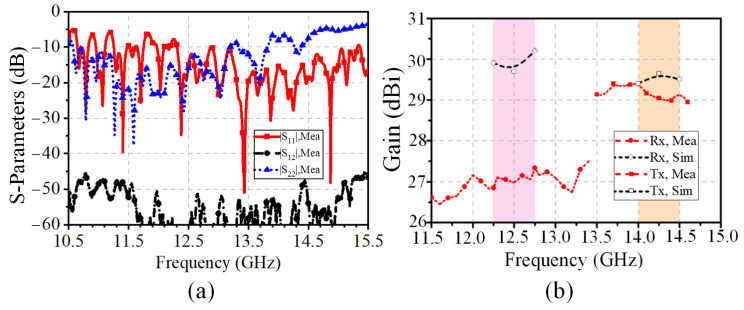
Measured and simulated results of the 16 × 24 antenna array: (**a**) S-parameters. (**b**) Gains in the normal direction.

**Figure 13:**
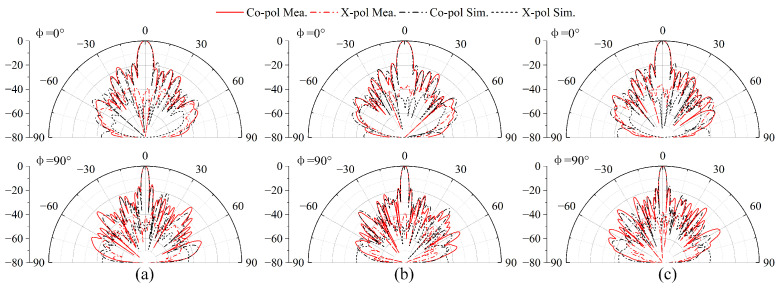
Simulated and measured radiation patterns of the 16 × 24 antenna array at the Rx-band: (**a**) 12.25 GHz. (**b**) 12.5 GHz. (**c**) 12.75 GHz.

**Figure 14:**
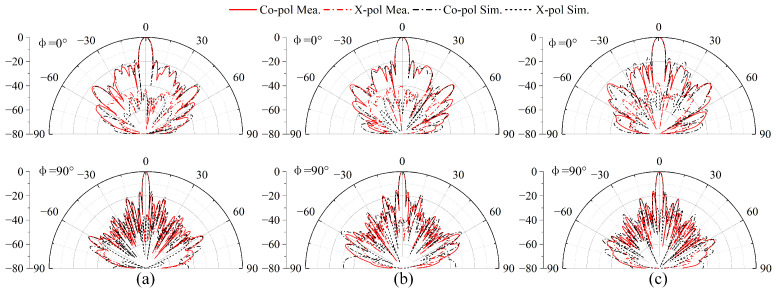
Simulated and measured radiation patterns of the 16 × 24 antenna array at the Tx-band: (**a**) 14 GHz. (**b**) 14.25 GHz. (**c**) 14.5 GHz.

The measured S-parameter results of the 16 × 24 antenna array are given in Figure 12a. The results show that the measured impedance bandwidths of the Rx and Tx ports are 22.1% (10.57–13.33 GHz) and 21.1% (12.31–15.32 GHz), respectively. The isolation is better than 47.5 dB in the Tx and Rx bands. In addition, the simulated and measured normalized radiation patterns at the Tx and Rx bands of the antenna array are shown in Figure 13 and Figure 14, respectively. It can be seen that the co-pol and cross-pol radiation patterns are highly satisfactory on both the *E*-plane and the *H*-plane. The measured cross-polarization level is below −38.3 dB at the boresight. The half-power beamwidths (HPWs) at the Rx band of the *H*-plane (φ = 0°) and *E*-plane (φ = 90°) are basically 5.9° and 3.2°, and the ones at the Tx band are 2.8° and 5.1°. Due to the non-uniform subarray scheme of the proposed 1D phased array, the sidelobes are suppressed. The first sidelobe level is less than −18 dB and −19 dB at the Rx and Tx bands, respectively.

**Figure 15:**
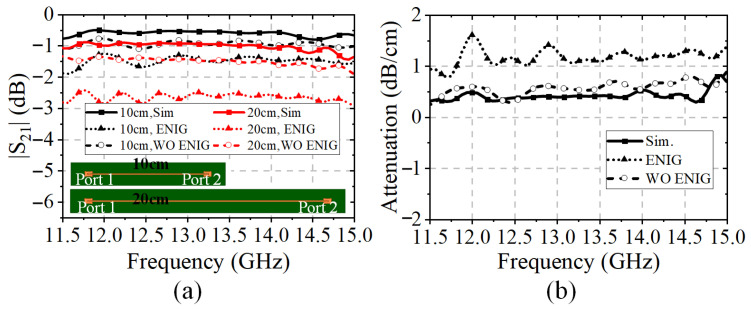
Analysis of the MS structure with and without the ENIG technology: (**a**) Simulated and measured S-parameters of the microstrip line with different lengths. (**b**) The attenuation of the microstrip line.

Furthermore, the simulated and measured results of the gains are illustrated in Figure 12b. It can be seen that the measured and simulated gains at the Tx-band are highly satisfactory. However, the simulated gain is 2.4 dB higher than the measured ones at 12.5 GHz. In fact, the difference is caused by the influence of surface metal coating during the fabrication. The comparison of the simulated and measured MS-line insertion loss with and without electroless nickel immersion gold (ENIG) technology is illustrated in Figure 15.

As shown in Figure 15b, the attenuation factor with the ENIG technology varies from 0.7 to 1.6 dB/cm throughout the Ku-band. Therefore, the ENIG technology for the dielectric substrates increases the attenuation of the microstrip feed-line and then decreases the measured gain performances.

## 4. The 32 × 24 Phased Array

**Figure 16:**
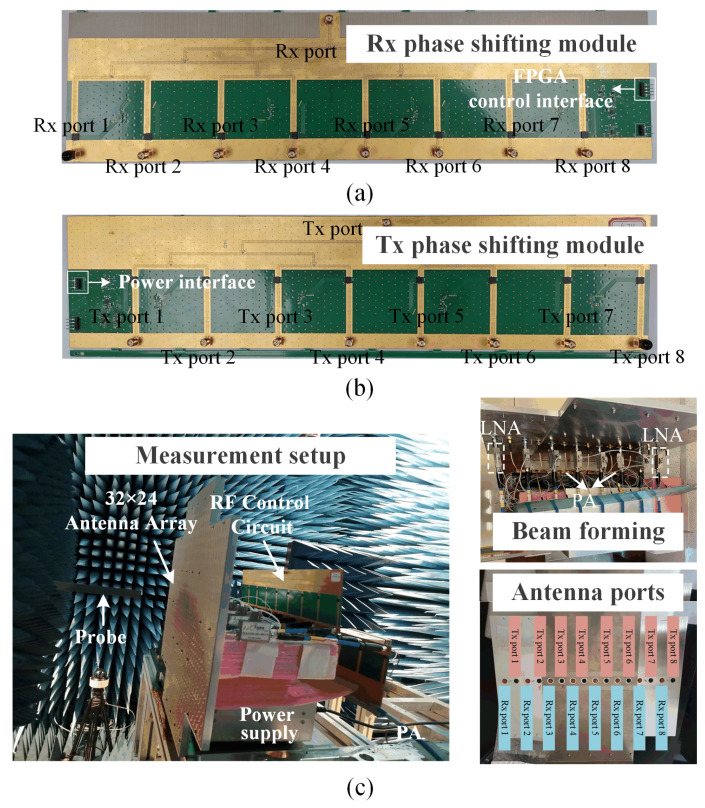
Photographs of the 32 × 24 phased array: (**a**) Rx phase-shifting module. (**b**) Tx phase-shifting module. (**c**) Measurement setup in the anechoic near-field chamber.

In this paper, a 32 × 24 antenna array is fabricated to validate the beam-scanning characteristic, whose configurations are presented in Figure 16. The 32 × 24 phased array is equivalent to doubling the 16 × 24 array in the *x*-direction. During the measurement process, each of the eight Rx ports is connected with low noise amplifiers (LNAs); then, eight coaxial cables for connecting the LNAs and the phase-shifting module are adopted. The Tx band ports are connected with eight phase-shifting channels separately, and the power amplifier (PA) is placed in front of the power divider for convenience.

**Figure 17:**
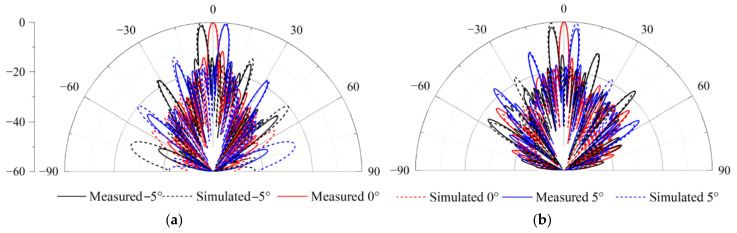
Simulated and measured scanning patterns of the 32 × 24 phased array: (**a**) Normalized radiation patterns at 12.5 GHz. (**b**) Normalized radiation patterns at 14.25 GHz.

**Figure 18:**
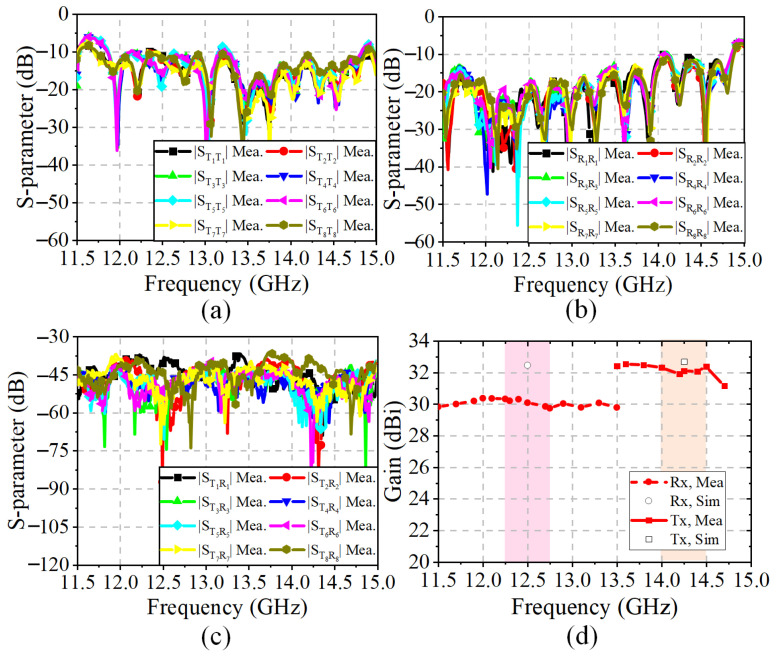
Simulated and measured results of the 32 × 24 phased array: (**a**) Reflection coefficients of the eight Tx ports. (**b**) Reflection coefficients of the eight Rx ports. (**c**) Isolation between the Tx and Rx ports. (**d**) Realized gains in the normal direction.

The beam-scanning normalized radiation patterns in the Rx and Tx bands are presented in Figure 17a,b. Due to the limited scanning angle requirements, the scanning range designed here is relatively small. The measurements show that the radiation pattern can be steered over the elevation range from −5° to 5°. The measured radiation patterns are in good agreement with the simulated ones. As shown in Figure 18a,b, the measured bandwidths of all the Rx and Tx ports are 26.1% (from 10.85 GHz to 14.1 GHz) and 11% (from 13.3 GHz to 14.85 GHz), respectively. The ports working in the Tx-band are greatly affected by the SMA welding; therefore, the S-parameters of these ports are inconsistent. Figure 18c shows that the isolations in the Rx band and Tx band are greater than 36.7 dB and 40 dB, respectively. In addition, the realized gains of the 32 × 24 antenna array are shown in Figure 18d with the elimination of the transmission losses of the coaxial line and connectors. Due to the limitation of the simulation environment, only the simulation results at the central frequencies (i.e., 14.25 GHz and 12.5 GHz) are available. The gain results are above 29.7 dBi across the Tx and Rx bands. The discrepancy between the simulated and measured gains is due to the interference of the surface metal coating technology on the microstrip feed line, which has been discussed in Section 3. The characteristics of low pointing error, wide bandwidth, and high isolation are particularly valuable for SATCOM beam-scanning applications.

Table 3 summarizes the comparison between the proposed phased array with the other published dual-polarized antenna arrays. Specifically, the antennas based on the waveguide in [13,14] have low attenuation and high-efficiency characteristics, whereas the problems of heavy weight, high cost, and low integrated level are non-negligible. The metasurface antenna in [16], with lightweight, low feed blockage loss and suitability for fabrication, is designed for the dual-polarized antenna. However, the large focal length-to-aperture diameter radio is not ideal for low-orbit spaceborne applications. Patch antennas with subarray structures are utilized to reduce the T/R RF modules [19,26]. Beam scanning up to 5° is achieved for the single linear-polarized radiation in [19]. The microstrip antenna in [26] is able to realize dual-polarization-switched beaming based on the Butler matrix (BM) with a complicated feeding network design. Compared with the same band patch antenna array in [27], the proposed antenna shows high low cross-polarization and isolation. In summary, the high gain, high isolation, low sidelobe levels, and stable radiation performances indicate that the proposed phased array antenna is valuable for low-orbit SATCOM applications.

## 5. Conclusions

For the characteristics of light weight, high integration, and high gain for LEO satellite communications, this paper proposes a Ku-band dual-polarized shared-aperture multilayer patch phased array based on PCB and CNC technology. The optimal non-uniform subarray scheme reduces the T/R modules and suppresses the sidelobe levels. In addition, the differential-fed SICL and MS architectures are responsible for the low cross-polarization and high gain features. Octagonal elements with Via-loaded rings are adopted for dual polarizations. To validate the beam-scanning characteristic, 32 × 24 phased arrays with T/R phase-shifting modules are fabricated. Measured flexible beam scanning, low pointing error, and stable radiation performances indicate the proposed antenna array is a good validation for phased array SATCOM.

## Figures and Tables

**Table 1:** Parameters of the element dimension (Unit: mm).

$h_{1}$	$h_{2}$	$h_{3}$	$h_{4}$	$h_{a1}$	$h_{a2}$	$h_{r}$	$l_{1}$	*d*	$l_{h1}$	$l_{h2}$	$l_{s1}$	$w_{s}$
0.762	0.508	0.508	0.508	2.5	4	2	5.9	2	1.3	1.2	3.1	0.2
$l_{o}$	$w_{1}$	$w_{2}$	$l_{2}$	$w_{100}$	$w_{70}$	$w_{100g}$	$w_{70g}$	$r_{2}$	$d_{x}$	$d_{y}$	$h_{\mathrm{pec}}$	
1.3	4.9	1.7	5.7	0.33	0.7	0.24	0.7	0.2	14	18.5	0.0175	

**Table 2:** Parameters of the feed networks dimension (Unit: mm).

$w_{50}$	$w_{z1}$	$w_{\mathrm{sp}1}$	$w_{\mathrm{sp}2}$	$w_{t2}$	$w_{t3}$	$w_{t4}$	$l_{t1}$
1.28	0.18	1.4	3.4	1.28	0.53	0.33	3.56
$l_{t2}$	$l_{t3}$	$l_{t4}$	$w_{50g}$	$r_{\mathrm{gz}1}$	$w_{\mathrm{gsp}1}$	$l_{\mathrm{gsp}1}$	$l_{\mathrm{gt}1}$
3.9	3.9	5.36	1.18	2.59	0.65	0.25	3.05
$l_{\mathrm{gt}2}$	$l_{\mathrm{gt}3}$	$l_{\mathrm{gt}4}$	$w_{\mathrm{gsp}2}$	$l_{\mathrm{gsp}2}$	$w_{\mathrm{sicl}}$		
3.4	3.4	3.45	1.4	0.7	2.5		

**Table 3:** Comparison between the proposed and reported dual-polarized antenna arrays.

Ref.	Structure	Element Number	$f_{0}$ (GHz)	BW	Gain (dBi)	SLL (dB)	CPL (dB)	Isolation (dB)
[13]	Air-filled waveguide	8 × 8	33	26.3%	28.5	−13	−30	36
[14]	Air-filled waveguide	4 × 4	12.5 14.25	8.9% 8.9%	26.51 26.48	−13	−32.9 −40.5	47.3 42
[16]	Transmitarray	788 × 777	12.5 14.25	4.2% -	30.2 32.3	−23 −22	−20 −26	>39.5
[19] *	patch metal	16 × 16	30	13.3%	28	−13	−22	n.a.
[26]	patch	4 × 4	28.7	19%	12	−8	−22	15
[27]	patch	8 × 8	12.5 14.5	28% 16.3%	19.33 20.11	−14 −12	−25 20	20
**This** **work**	**patch** **+metal**	**16** × **24**	**12.25** **14.5**	**22.1%** **21.1%**	**27.3** **29.4**	**−18** **−19**	**−38.3** **−38.6**	**52** **47.5**
		**32** × **24**	**12.25** **14.5**	**26.1%** **11%**	**30.3** **32.3**	**−19** **−19**	**−30.5** **−28.3**	**36.7** **39.9**

* Single linear-polarization array. + Without the feeding structure.

## Data Availability

The data presented in this study are available on request from the corresponding author. The data are not publicly available due to privacy considerations.

## Abbreviations

The following abbreviations are used in this manuscript:

T/R	transmitter and receiver
SICL	substrate integrated coaxial line
MS	microstrip
SATCOM	satellite communication
